# Glucagon-like peptide-1 receptor-agonists treatment for cardio-metabolic parameters in schizophrenia patients: a systematic review and meta-analysis

**DOI:** 10.3389/fpsyt.2023.1153648

**Published:** 2023-05-05

**Authors:** Abdulrhman Khaity, Nada Mostafa Al-dardery, Khaled Albakri, Omar A. Abdelwahab, Mahmoud Tarek Hefnawy, Yaman A. S. Yousef, Ruaa E. Taha, Sarya Swed, Wael Hafez, Rene Hurlemann, Mohamed E. G. Elsayed

**Affiliations:** ^1^Faculty of Medicine, Elrazi University, Khartoum, Sudan; ^2^Faculty of Medicine, Fayoum University, Fayoum, Egypt; ^3^Faculty of Medicine, The Hashemite University, Zarqa, Jordan; ^4^Faculty of Medicine, Al-Azhar University, Cairo, Egypt; ^5^Faculty of Medicine, Zagazig University, Zagazig, Egypt; ^6^Faculty of Medicine, Khartoum University, Khartoum, Sudan; ^7^Faculty of Medicine, Aleppo University, Aleppo, Syria; ^8^NMC Royal Hospital, Khalifa City, Abu Dhabi, United Arab Emirates; ^9^Medical Research Division, Department of Internal Medicine, The National Research Centre, Cairo, Egypt; ^10^Department of Psychiatry, School of Medicine and Health Sciences, Carl von Ossietzky University Oldenburg, Oldenburg, Germany; ^11^Department of Psychiatry, University Hospital Bonn, Bonn, Germany; ^12^Research Center Neurosensory Science, Carl von Ossietzky University Oldenburg, Oldenburg, Germany; ^13^Department of Psychiatry and Psychotherapy III, University of Ulm, Ulm, Germany

**Keywords:** schizophrenia, systematic review, meta-analysis, GLP-1 receptor agonists, treatment

## Abstract

**Aims:**

We performed this meta-analysis to evaluate the efficacy and safety of glucagon-like peptide-1 receptor-agonists (GLP-1RA) treatment on cardio-metabolic parameters among antipsychotic-treated patients with schizophrenia.

**Methods:**

We searched the Web of Science, Cochrane Central Register of Controlled Trials, PubMed, PsycINFO, and Scopus for relevant Randomized Clinical trials (RCTs) from inception until 1 August 2022. Documents were screened for qualified articles, and all concerned outcomes were pooled as risk ratios (RR) or mean difference (MD) in the meta-analysis models using Review Manager (RevMan version 5.4).

**Results:**

Pooling data from 7 RCTs (398 patients) showed that GLP-1 RA was superior to placebo with regard to body weight [MD = - 4.68, 95% CI (-4.90,−4.46), *P* < 0.00001], waist circumference [MD = - 3.66, 95% CI (-3.89,−3.44), *P* < 0.00001], body mass index (BMI) [MD = - 1.09, 95% CI (-1.25,−0.93), *P* < 0.00001], systolic blood pressure (SBP) [MD = - 3.07, 95% CI (-3.61,−2.53), *P* < 0.00001], and diastolic blood pressure (DBP) [MD = - 2.02, 95% CI (-2.42,−1.62), *P* < 0.00001]. The total effect did not favor either of the two groups with respect to insulin and respiratory adverse events {[MD = - 0.06, 95% CI (-0.36, 0.24), *p* = 0.70], [RR = 0.66, 95% CI (0.31, 1.40), *p* = 0.28]; respectively}.

**Conclusion:**

Our analysis revealed that GLP-1 RA treatment is safe and effective on cardio-metabolic parameters over control in antipsychotic-treated patients with schizophrenia. Nevertheless, the present evidence is not sufficient to confirm the safety and efficacy of GLP-1RA treatment on insulin and respiratory adverse events. Therefore, further studies are recommended.

**Systematic review registration:**

http://www.crd.york.ac.uk/PROSPERO/, identifier: CRD42022333040.

## 1. Introduction

Schizophrenia is one of the mental conditions that affect how individuals think, act, express emotions, interpret reality, and interact with others (1). A combination of antipsychotics and psychosocial therapies has a crucial role in improving a schizophrenia patient's life (2). However, several factors, including genetic susceptibility to diabetes, limited physical exercise, the use of antipsychotic drugs, and malnutrition, raise the risk of cardiometabolic illness in patients with schizophrenia (3–5).

It is worth mentioning that antipsychotic treatments may cause obesity, olanzapine along with clozapine have the highest tendency for weight increase (6). Metabolic syndrome has been detected in half of the schizophrenia patients who received clozapine and one-third of those taking olanzapine (3). The evidence for therapies targeting antipsychotic-associated obesity is scant at present and still does not achieve the target effect (7). This could be elucidated by poor exposure to physical exercise programs. In addition, no positive physical health impacts were determined for dextroamphetamine, famotidine, ranitidine, orlistat, or fluoxetine (8). Moreover, sibutramine was taken off the market due to cardiovascular risks, whereas rimonabant was taken off the market due to an elevated risk of developing depression, anxiety, and suicidal thought (9, 10).

Due to these limitations, attention to glucagon-like peptide-1 receptor agonists (GLP-1RAs) arose to combat the weight increase observed with antipsychotic medication, especially clozapine and olanzapine (11, 12). GLP-1RA has been demonstrated to decrease blood glucose as well as body weight in persons with and without type 2 diabetes (12, 13). Additionally, GLP-1RA therapy reduces the risk of significant adverse cardiovascular consequences (14).

Regarding the mechanism of action, GLP-1RA activate the corresponding receptors in the pancreas, leading to enhanced release of insulin and inhibited glucagon release responses (15, 16). Their action on GLP-1 receptors in the central nervous system and gastrointestinal tract causes reduced appetite and delayed absorption of glucose due to slower gastric emptying (16, 17). In addition, they slow the process of gastric emptying, limiting significant post-meal glycemic levels (15). The short-acting agents (exenatide b.i.d., lixisenatide) have limited effect on fasting and overnight plasma glucose levels, but they maintain their influence on gastric emptying during long-term treatment (15). On the other hand, the long-acting GLP-1 receptor agonists (liraglutide, dulaglutide, exenatide, semaglutide, and albiglutide) have more profound effects on fasting and overnight plasma glucose levels, and HbA1c (15).

Previous studies evaluated the efficacy of GLP-1RAs for antipsychotic-associated cardio-metabolic risk factors. They found that patients with schizophrenia may benefit from GLP-1RA medication for weight control. However, these studies have shown conflicting findings regarding the impact of GLP-1RA on blood pressure, HbA1C, total cholesterol, and serious adverse events (18, 19). Therefore, this meta-analysis was conducted to precisely investigate the efficacy and safety of GLP-1RAs on cardio-metabolic parameters among antipsychotic-treated schizophrenic patients.

## 2. Methods

We tracked the Cochrane Collaboration recommendations and PRISMA guidelines to prepare this meta-analysis (20). All steps of this study were prespecified and documented at the (PROSPERO): CRD42022333040.

### 2.1. Criteria for study eligibility

The following conditions were considered for the study:

1) Design: studies that were designated as randomized clinical trials (RCTs).

2) Population: studies that enrolled schizophrenia or schizoaffective disorder patients who were administrated antipsychotic treatments and were overweight or obese or prediabetes.

3) Intervention: studies where the intervention was subcutaneously injection of GLP1-RA whether once-weekly exenatide or once-daily liraglutide. Among the included studies, the tolerability of liraglutide was recorded by dose escalation pattern, up titration of 0.6 mg per week to a daily dose of 3.0 mg. Participants who did not tolerate up titration continued on the highest tolerable dose. Regarding exenatide, the tolerability was measured by monitoring for adverse effects at trial visits.

4) Comparator: studies that used a placebo as a comparator were eligible.

5) Outcomes: studies stated one or more of the following outcomes: body weight, metabolic syndrome parameters [blood pressure (BP), waist circumference, and fasting plasma glucose (FPG)], and adverse drug reaction. In addition, body mass index (BMI), HbA1c, insulin, and bone turnover markers, including Procollagen type I N-terminal propeptide (PINP) level and C-terminal cross-linking telopeptide of type I collagen (CTX) level.

We excluded articles that were not in English, conference abstracts, single-arm, observational, quasi-clinical trials, and studies that used other types of GLP-1 receptor agonists.

### 2.2. Electronic searches

A comprehensive literature search was conducted of five electronic databases (Web of Science, PubMed, Cochrane Library for clinical trials in CENTRAL, Scopus, and PsycINFO via Ovid) from inception till 1 August 2022 using the following query: [((exenatide) OR (Liraglutide) OR (Victoza) OR (Saxena) OR (Glucagon-like peptide-1 receptor agonist) OR (GLP-1RA) AND (schizophrenia)) AND (antipsychotic)].

### 2.3. Study selection process

The titles and abstracts of all citations considered for inclusion were reviewed by three authors independently. Then, we extracted the full text of the selected studies to evaluate their applicability and validated them according to our systematic review and meta-analysis standards. Discrepancies were resolved by consensus.

### 2.4. Data extraction

Four reviewers carried out the extraction of data independently using a uniform sheet. From each included trial, the following data were extracted: (1) characteristics of study design, (2) characteristics of the study population, (3) risk of bias scopes (4) study outcomes: body weight, metabolic syndrome parameters [blood pressure (BP), waist circumference, and fasting plasma glucose (FPG)], and adverse drug reaction. In addition, BMI, HbA1c, insulin, and bone turnover markers, including PINP and CTX levels.

### 2.5. Quality appraisal

The quality of each involved trial was evaluated precisely by two authors independently. We utilized a table of the quality appraisal in the Cochrane handbook (part 2, chapter 8.5). The Cochrane tool for evaluating the possibility of bias comprises the subsequent areas: (1) Random sequence generation (selection bias), (2) allocation concealment (selection bias), (3) blinding of participants and personnel (performance bias), (4) blinding of outcome assessment (detection bias), (5) incomplete outcome data (attrition bias), (6) selective reporting (reporting bias) and (7) other potential sources of bias. The authors' decision is classified as Unclear risk, Low risk, or High risk of bias. The conflicts were solved by the third author.

### 2.6. Statistical analysis

Mean changes from baseline for body weight, metabolic syndrome parameters, BMI, HbA1c, and insulin, were pooled as mean difference (MD) between the two groups from baseline to the endpoint in the meta-analysis models utilizing the inverse variance (IV) method. We assumed a fixed-effect model of the MD as the main analysis model. At the same time, relative risk (RR) was used to pool dichotomous data in a fixed-effect model using the Mantel-Haenszel (M-H) method. RevMan software (version 5.4 for Windows) was applied to run the statistical analysis. In addition, we used the Chi-square test (Cochrane Q test) to assess the statistical heterogeneity of the included studies. Significant heterogeneity was reflected by I-square > 50% with a *P*-value < 0.1.

### 2.7. Certainty assessment

We performed sensitivity analysis “leave-one-out meta-analysis” for each outcome in the meta-analysis in multiple scenarios to test the strength of the evidence and ensure that the total effect size was not reliant on any certain individual study. In case of significant statistical heterogeneity among included studies (i.e., variability beyond what would be expected by chance), that is may indicate that the studies are too dissimilar to combine and we excluded them from the analysis.

## 3. Results

### 3.1. Results of study selection and characteristics

**Figure 1:**
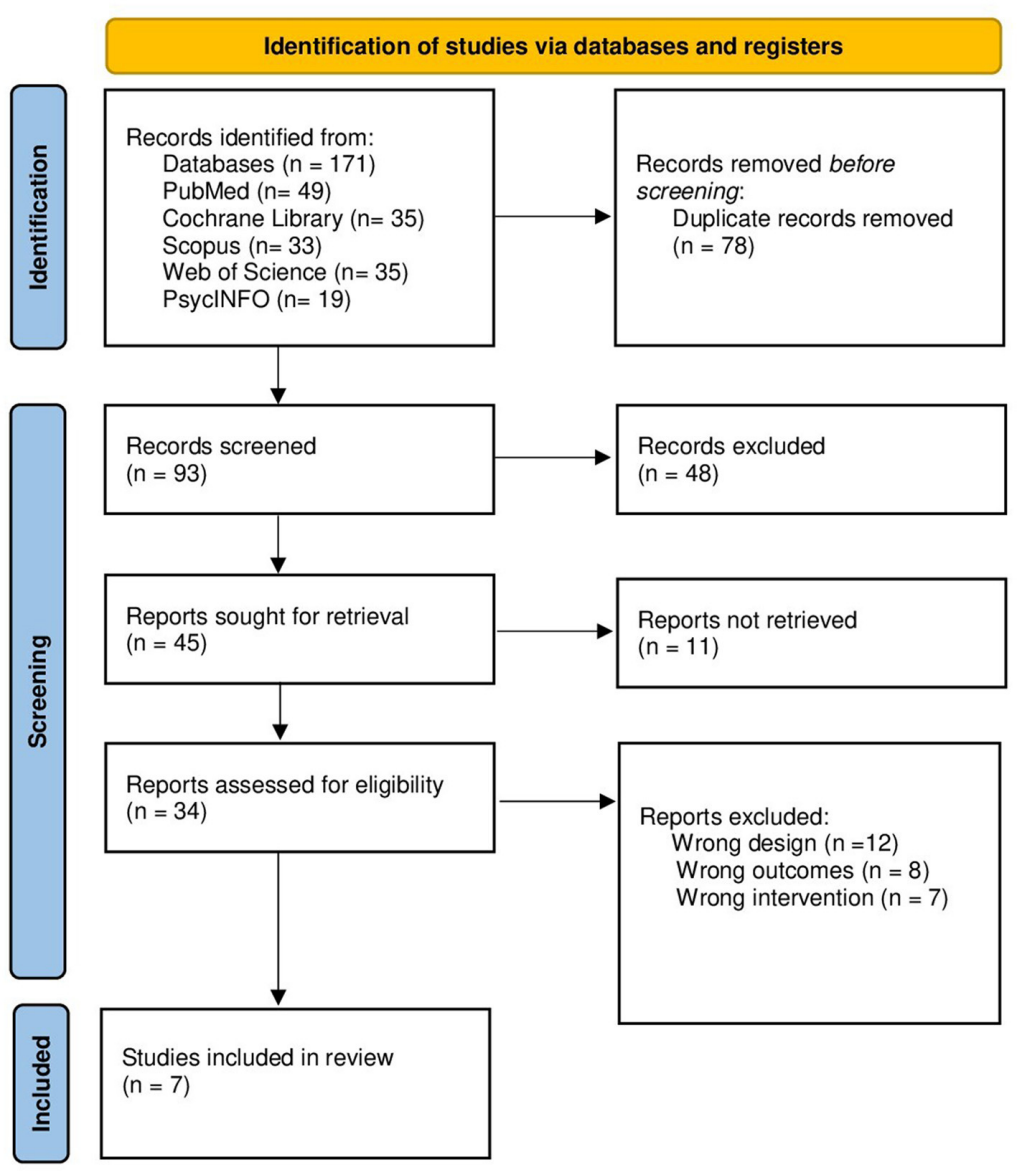
PRISMA flow diagram of studies' screening and selection.

Our search strategy yielded 171 articles. After removing duplication using Endnote X8.0.1., 93 abstracts were evaluated, and 34 articles were suitable for full-text screening. During the full-text evaluation, 27 studies were left out, 12 of them were excluded due to discrepancies in design with our study and eight articles were excluded because they neither meet the primary nor secondary outcomes of this study. Moreover, we excluded seven studies because they involved different interventions. Finally, we included 7 RCTs (398 patients) in this systematic review and meta-analysis (21–27), Four of them used liraglutide and the last three studies rely on exenatide. The PRISMA flow diagram of the study selection process is shown in Figure 1. A summary of the included articles, their design, and their findings are demonstrated in Table 1.

**Table 1:** Summary of the included studies.

**Study ID**	**Study design**	**Country**	**Participants**	**Groups**		**Sample Size**		**Duration of the treatment (weeks)**	**Results**
				**Intervention**	**Control**	**Intervention**	**Control**		
Eriksson et al. (21)	Double-blind, randomized, placebo-controlled trial	Denmark	The participant's age was between 18 and 65 years old. At least one of the antipsychotic drugs was received by the people in this study. They experienced obesity with a BMI ≥ 30 kg/m2. However, the participants in this study were clinically stable schizophrenia spectrum.	Exenatide	Placebo	20	20	12	The bone markers did not experience a significant change in schizophrenia patients. This study's results indicated no noticeable difference between the reference population and sampled one regarding bone status.
Ishøy et al. (22)	Randomized, Placebo-controlled Trial	Denmark	The participants were between 18 and 65 years old of age. Obesity with a BMI ≥ 30 kg/m2 were detected among the population. They also are on the antipsychotic drug and had a clinically stable schizophrenia spectrum.	Exenatide	Placebo	20	20	12	Exenatide treatment did not contribute to weight loss when used by antipsychotic-treated patients who are diagnosed with schizophrenia.
Larsen et al. (23)	Randomized Clinical Trial	Denmark	The participants' ages were between 18 to 65 years old with a BMI of 27 or greater, they were also diagnosed with a schizophrenia spectrum disorder, and received stable treatment with clozapine or olanzapine.	Liraglutide	Placebo	47	50	16	The liraglutide group witnessed a noticeable decrease in the following measures compared to the placebo group: body weight, waist circumference, systolic blood pressure, visceral fat, and LDL levels. In addition, the liraglutide also showed side effects in the GIT mainly.
Maagensen et al. (24)	Randomized controlled trial	Denmark	The participants are diagnosed with schizophrenia or psychosis, are treated with clozapine or olanzapine, and have an IBM of 27 or greater.	Liraglutide	Placebo	35	37	16	The results after 16 weeks of the treatment did not show any differences between the groups in bone turnover markers.
Siskind et al. (25)	Randomized controlled trial	Australia	The participant's ages were between 18 and 64, with an IBM of 30 to 45, and were diagnosed with schizophrenia or schizoaffective disorder. They were also been taking oral clozapine for more than 18 weeks.	Exenatide	Placebo	14	14	24	After completing the 28 weeks of treatment, patients treated with Exenatide experienced a higher weight loss mean compared to the placebo group. Moreover, the BMI indicator, HbA1c, and fasting blood glucose showed a noticeable lowering in the treated group, compared to the placebo. Other metabolic components did not show any remarkable differences.
Svensson et al. (26)	Randomized, double-blinded, placebo-controlled trial	Denmark	The participant's ages were between 18 and 65 years old and diagnosed with schizophrenia-spectrum disorder. These participants were treated with clozapine or olanzapine for at least 6 months, BMI ≥27.	Liraglutide	Placebo	41	46	16	The reduction of body weight remained sustained after one year of stopping Liraglutide treatment (the follow-up duration). Nevertheless, the “metabolic parameters” such as fasting glucose, lipid profile, C-peptide, and glycated hemoglobin, all their levels had regressed to the base of the baseline after one year of stopping the liraglutide treatment
Whicher et al. (27)	Double-blind, randomized, placebo-controlled pilot trial	The United Kingdom	The participant's ages were between 18 to 75 years old with an IBM ≥27, diagnosed with schizophrenia or schizoaffective disorder or first-episode psychosis, and had been prescribed antipsychotic medication for at least 1 month.	Liraglutide	Placebo	15	19	24	For the participants who received the liraglutide, an obvious reduction in body weight was observed, compared to the placebo group. Also, the intervention group results showed a reduction in waist circumference, BMI, and HbA1c levels.

The basic characteristics of the included studies revealed that all ages of participants ranged from 18 to 75 years old (mean age = 42.3 years, SD = 10.7). Most of the participants were male 63.3%. Additionally, the mean BMI of the included population was 36.1 kg/m2 (SD 6.3). All the participants in the included studies experienced schizophrenia. More information regarding the baseline characteristics of the included articles is shown in Table 2.

**Table 2:** Baseline characteristics of the included studies.

**Study ID**	**Groups**	**Age Mean (SD)**	**Gender (Male) *N* (%)**	**Race N (%)**		**Diagnosis *N* (%)**	**Smoking *N* (%)**	**BMI (kg/m2) Mean (SD)**	**Height (m) Mean (SD)**	**Weight (Kg) Mean (SD)**	**Waist circumference (cm) Mean (SD)**	**SBP (mmHg) Mean (SD)**	**DBP (mmHg) Mean (SD)**	**Fasting plasma glucose (mmol/L) Mean (SD)**	**HbA1c (mmol/mol) Mean (SD)**	**Fasting lipids (mmol/L) Mean (SD)**				**Type of the Antipsychotic medication used by participants** ***N*** **(%)**				
					***Schizophrenia***	***Schizoaffective disorder***										**Total cholesterol**	**HDL cholesterol**	**LDL cholesterol**	**Triglycerides**	**Clozapine**	**Olanzapine**	**Risperidone**	**Flupenthixol**	**Multiple antipsychotic medication**
Eriksson et al. (21)	Exenatide	37.1 (10.6)	11 (48)	Caucasian 21 (91) Mongolian 2 (9)	21 (91)	2 (9)	7 (30)	39.2(3.8)	NR	117.1 (16.7)	NR	NR	NR	NR	NR	NR	NR	NR	NR	NR	NR	NR	NR	7 (30)
	Placebo	34.5(10.1)	10 (45)	Caucasian 19 (86) Mongolian 3 (14)	20 (91)	2 (9)	1 (5)	38.4(6.1)	NR	110.6 (17.7)	NR	NR	NR	NR	NR	NR	NR	NR	NR	NR	NR	NR	NR	10 (45)
Ishøy et al. (22)	Exenatide	37.4 (10.7)	11(55)	Caucasian 47.5% Mongolian 2.5%	NR	NR	7 (35)	39.5 (3.5)	172.8 (8.4)	118.3 (16.0)	128.4 (11.1)	122.4 (14.6)	83.7 (12.4)	5.1 (0.3)	33.3 (3.3)	4.5 (1.0)	0.9 (0.2)	2.6 (0.9)	1.5 (0.7)	NR	NR	NR	NR	NR
	Placebo	34.4 (10.6)	9(45)	Caucasian 45.0% Mongolian 5.0%	NR	NR	1 (5)	38.6 (6.3)	170.4 (10.1)	111.7 (18.0)	125.3 (13.5)	110.5 (8.9)	78.1 (9.5)	5.3 (0.5)	34.7 (4.8)	2.4 (0.5)	1.0 (0.3)	2.4 (0.5)	1.4 (0.8)	NR	NR	NR	NR	NR
Larsen et al. (23)	Liraglutide	42.1 (10.7)	30 (63.8)	NR	46 (97.9)	0	NR	33.7 (5.1)	NR	103.3 (16.1)	117.3 (12.4)	125.9 (10.5)	84.3 (9.8)	100.9 (18.0)^**^ mg/dl	5.6 (0.4)	240.6 (69.5)^**^, mg/dl	42.5(19.3)^**^, mg/dl	123.5(57.9)^**^, mg/dl	177.0 (150.4)^**^ mg/dl	32(68.1)	15(31.9)	NR	NR	0
	Placebo	43.0 (10.5)	30 (60.0)	NR	50 (100)	0	NR	33.9 (6.6)	NR	102.4 (23.9)	115.9 (15.1)	125.2 (14.1)	84.6 (7.6)	102.7(16.2)^**^, mg/dl	5.5 (0.4)	196.9(42.5)^**^, mg/dl	42.5(23.2)^**^, mg/dl	127.4(42.5)^**^ mg/dl	177.0 (123.9)^**^ mg/dl	41(82)	6(12)	NR	NR	3(6)
Maagensen et al. (24)	Liraglutide	43.9 (10.4)	24 (69)	NR	NR	NR	NR	33.3 (4.9)	NR	103.2 (16.0)				5.7 (0.57)	36.8 (4.7)	NR	NR	NR	NR	22 (63)	13 (37)	NR	NR	0 (0)
	Placebo	42.4 (9.8)	25 (68)	NR	NR	NR	NR	33.7 (6.7)	NR	103.6 (24.9)				5.8 (0.63)	36.2 (3.4)	NR	NR	NR	NR	31 (84)	3 (8)	NR	NR	3 (8)
Siskind et al. (25)	Exenatide	(18-64)^*^	11 (61.1)	NR	NR	NR	NR	35.56 (2.42)	1.74 (0.07)	108.0 (8.8)	118.43 (8.30)	122 (7)	82 (7)	5.5 (1.0)	5.4 (0.5)	NR	1.00 (0.26)	NR	2.89 (1.72)	NR	NR	NR	NR	NR
	Placebo		7 (50)	NR	NR	NR	NR	35.80 (3.77)	1.69 (0.09)	102.7 (17.3)	115.61 (11.84)	124 (8)	79 (9)	5.4 (1.0)	5.5 (0.7)	NR	1.12 (0.42)	NR	2.74 (1.72)	NR	NR	NR	NR	NR
Svensson et al. (26)	Liraglutide	42 (10.7)	30 (63.8)	NR	46 (97.9)	NR	NR	33.7 (5.1)	NR	103.3 (16.1)	117.3 (12.4)	125.9 (10.5)	84.3 (9.8)	5.6 (6.3)^**^	37.5 (4.7)	5.3 (6.2)^**^	3.3 (1.1)	1.1 (1.2)^**^	2 (1.94)^**^	31 (66)	15 (31.9)	NR	NR	NR
	Placebo	43 (10.5)	30 (60)	NR	50 (100)	NR	NR	33.9 (6.6)	NR	102.4 (23.9)	115.9 (15.1)	125.2 (14.1)	84.6 (7.6)	5.7 (6.2)^**^	37 (4)	5.1 (5.6)^**^	3.1 (0.9)	1 (0.9)^**^	2 (1.57)^**^	40 (80)	6 (12)	NR	NR	NR
Whicher et al. (27)	Liraglutide	42.7 (11.3)	15 (62)	White British, Irish, other 21 (88%) Another ethnic group 3 (13%)	15 (62)	8 (33)	13 (62)	37.5 (6.9)	1.72 (0.13)	111.4 (25.5)	123.8 (20.1)	130 (24)	92 (23)	5.2 (0.5)	37 (6)	5.0 (1.0)	1.3 (0.3)	3.1 (0.8)	1.8 (0.8)	3 (12%)	2 (8%)	1 (4%)	4 (17%)	5 (21%)
	Placebo	45.4 (10.7)	9 (39)	White British, Irish, other 17 (74%) Another ethnic group 6 (26%)	13 (56%)	9 (39%)	7 (32)	41.0 (6.7)	1.71 (0.11)	117.7 (23. 5)	130.6 (14.0)	134 (15)	93 (7)	5.1 (0.7)	40 (5)	5.0 (1.2)	1.4 (0.3)	2.7 (1.0)	1.8 (0.8)	6 (26%)	0 (0%)	2 (9%)	3 (13%)	3 (13%)

* Data presented in range;

** Data presented in median (IQR); NR, Not Reported.

### 3.2. Risk of bias assessment

**Figure 2:**
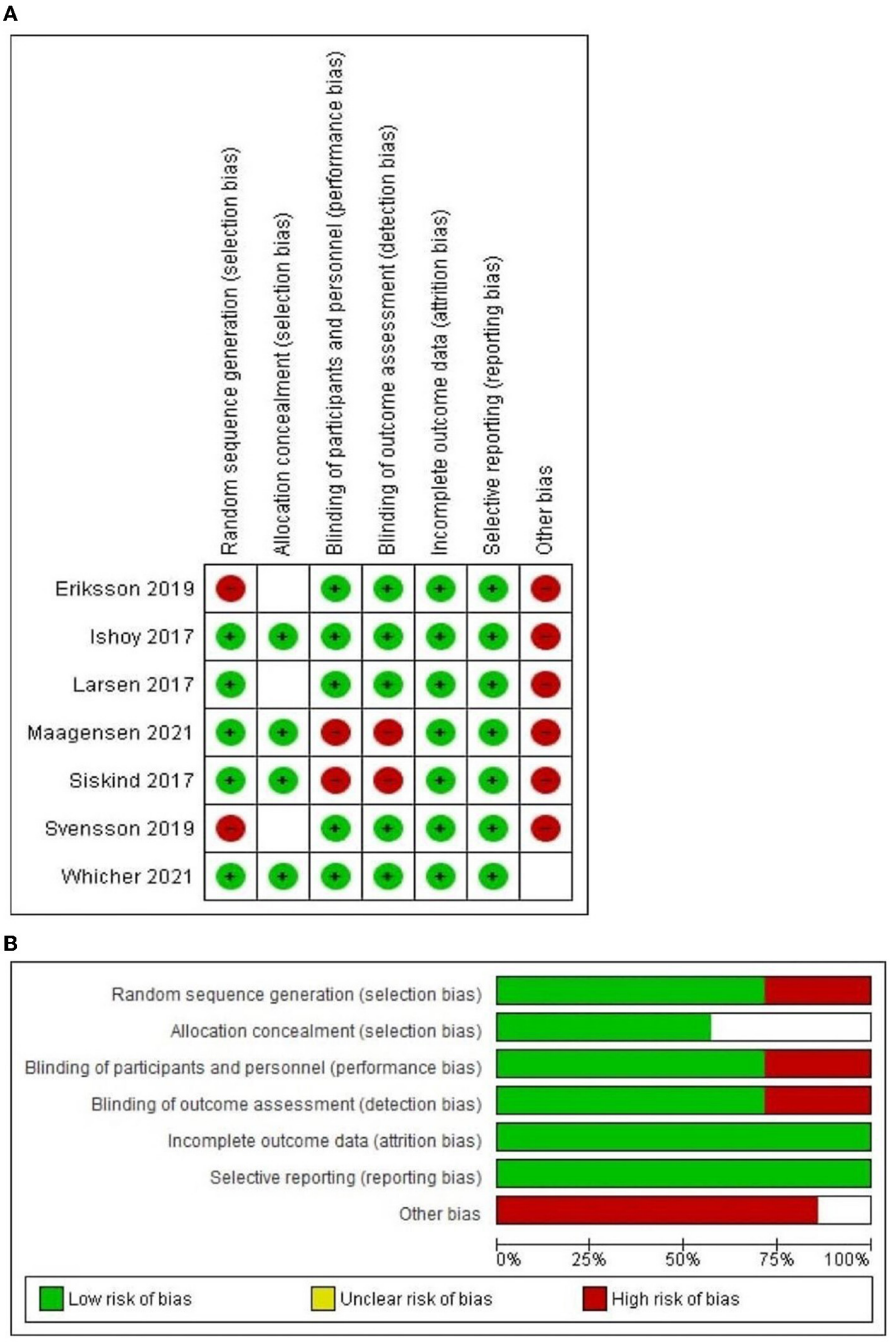
**(A)** The risk of bias summary graph according to Version 2 of the Cochrane risk-of-bias tool for randomized trials (RoB2); **(B)** The risk of bias traffic light graph according to Version 2 of the Cochrane risk-of-bias tool for randomized trials (RoB2).

The quality of included articles ranged from fair to poor quality by the Cochrane Risk of Bias assessment tool for RCTs, Figures 2A, B reveals an overview of the Cochrane Risk of Bias score per item; red color for high-risk, green color for low risk and white for unclear.

### 3.3. Outcomes

#### 3.3.1 Body Weight

**Figure 3:**
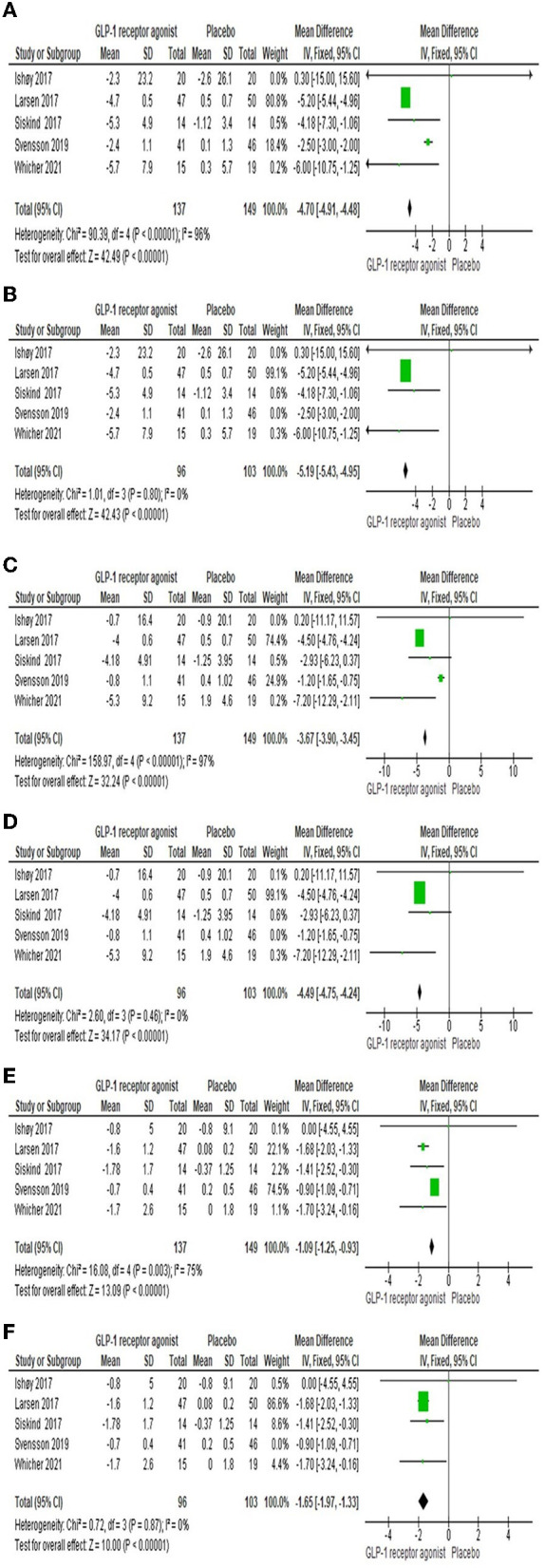
Forest plots of mean difference in **(A)** body weight, **(B)** overall body weight after sensitivity analysis, **(C)** Waist circumference, **(D)** Waist circumference after sensitivity analysis, **(E)** BMI, and **(F)** BMI after sensitivity analysis.

Five studies (22, 23, 25–27) reported the efficacy of GLP-1RA on body weight compared to placebo. The findings presented that there was a significant difference between the two groups [MD = −4.7 (-4.91,−4.48), *P*-value < 0.00001] as shown in Figure 3A. The pooled studies were heterogeneous (*P*-value < 0.00001, *I*^2^ = 96%). After performing the sensitivity analysis and excluding Svensson et al. (26), the heterogeneity was resolved (*P*-value = 0.8, *I*^2^ = 0%). The results are still significant and prefer the experimental group [MD = −5.19 (-5.43,−4.95), *P*-value < 0.00001] as shown in Figure 3B.

#### 3.3.2 Waist circumference

Waist circumference was investigated by five studies (22–26). There was a significant difference among both groups [MD = −3.67 (-3.9,−3.45), *P*-value < 0.00001], but there was a heterogeneity between the studies (*P*-value < 0.00001, *I*^2^ = 97%) as shown in Figure 3C. However, sensitivity analysis resolved the heterogeneity; we excluded Svensson et al. (26), as shown in Figure 3D.

#### 3.3.3 BMI

Five studies (22–26) reported the effect of GLP-1RA on the BMI compared to placebo. The consequences revealed that there was a significant difference among both groups [MD = −1.09 (-1.25,−0.93), *P*-value < 0.00001] as shown in Figure 3E. The pooled studies were heterogeneous (*P*-value = 0.003, *I*^2^ = 75%). After performing the sensitivity analysis and excluding Svensson et al. (26), the heterogeneity was resolved (*P*-value = 0.87, *I*^2^ = 0%). The results are still significant and prefer the experimental group [MD = −1.65 (-1.97,−1.33), *P*-value < 0.00001] as shown in Figure 3F.

#### 3.3.4 Blood pressure

**Figure 4:**
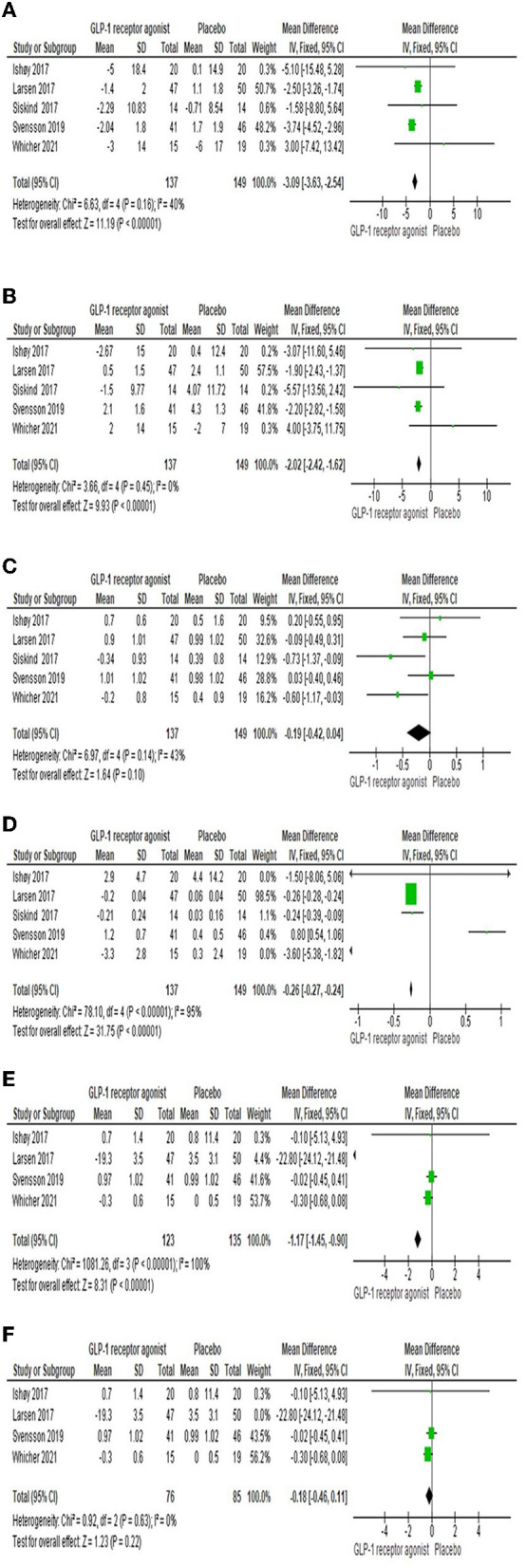
Forest plots of mean difference in **(A)** systolic blood pressure, **(B)** diastolic blood pressure, **(C)** FBG, **(D)** HbA1c, **(E)** Total cholesterol, and **(F)** Total cholesterol after sensitivity analysis.

Five studies (22–26) assessed the systolic and diastolic blood pressures, and the overall MD showed the GLP-1RA group was significantly lower than control group [MD = −3.09 (-3.63,−2.54), *P-*value < 0.00001; MD = −2.02 (-2.42,−1.62), *P-*value < 0.00001], and the pooled articles were homogenous (*P*-value = 0.16, *I*^2^ = 40%; *P*-value = 0.45, *I*^2^ = 0%) as shown in Figures 4A, B, respectively.

#### 3.3.5 Fasting plasma glucose

The impact of the GLP-1RA on fasting plasma glucose was examined by five studies (22–26). Results showed that the total effect did not favor either of two groups [MD = −0.19 (-0.42, 0.04), *P*-value = 0.1]. The findings of the studies were homogenous (*P*-value = 0.14, *I*^2^ = 43%) as shown in Figure 4C.

#### 3.3.6 HbA1c

Five studies (22–26) investigated the HbA1c, and the overall MD revealed that there was a significant difference among the two groups [MD = −0.26 (-0.27,−0.24), *P*-value < 0.00001], but there was heterogeneity between the studies (*P*-value < 0.00001, *I*^2^ = 95%) as shown in Figure 4D.

#### 3.3.7 Total cholesterol

Total cholesterol was investigated by four studies (22–25). There was a significant difference among the two groups [MD = −1.17 (-1.45,−0.9), *P*-value < 0.00001], but there was a heterogeneity between the studies (*P*-value < 0.00001, *I*^2^ = 100%) as shown in Figure 4E. However, sensitivity analysis resolved the heterogeneity; we excluded Larsen et al. (23), as shown in Figure 4F.

#### 3.3.8 Insulin level

**Figure 5:**
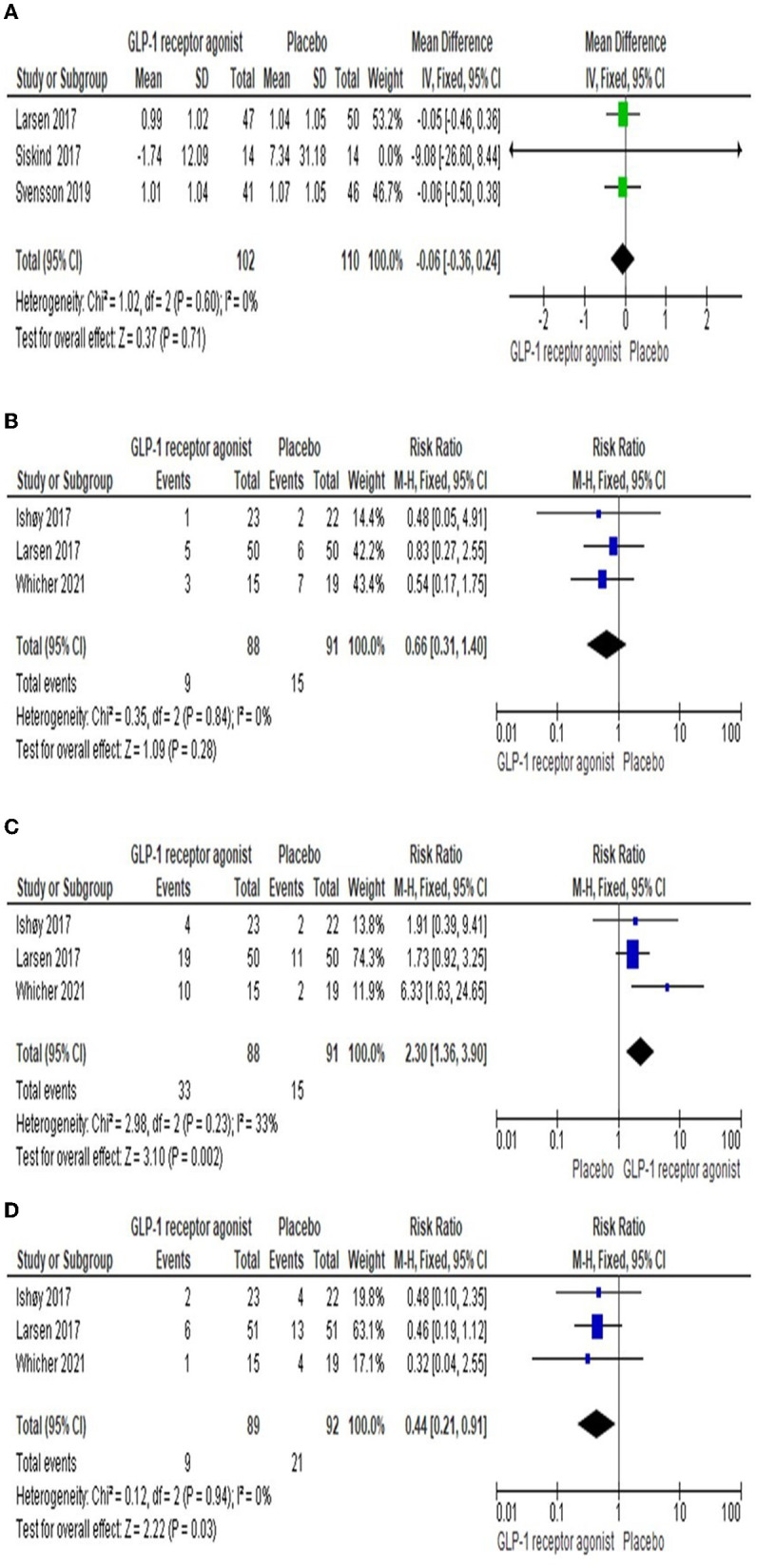
Forest plots of mean difference in **(A)** Insulin level. In addition, Forest plots of risk ration in **(B)** Respiratory side effects, **(C)** Gastrointestinal side effects, and **(D)** Serious side effects).

Three studies (23, 25, 26) reported the impact of GLP-1RA on the insulin level compared to Placebo. The findings revealed that there was no significant difference observed among both groups [MD = −0.06 (-0.36, 0.24), *P*-value = 0.71]. The pooled articles were homogenous (*P*-value = 0.60, *I*^2^ = 0%) as shown in Figure 5A.

#### 3.3.9 Respiratory side effects

Three studies (22, 23, 27) reported the number of cases suffered from respiratory side effects, including upper respiratory tract infection and asthma, in each group. The overall RR demonstrated that there was no significant difference between the two groups [RR = 0.66 (0.31, 1.4), *P*-value = 0.28]. The pooled studies were homogenous (*P*-value = 0.84, *I*^2^ = 0%) as shown in Figure 5B.

#### 3.3.10 Gastrointestinal adverse events

Gastrointestinal adverse events, including nausea, vomiting, constipation, and diarrhea, were observed in three studies (22, 23, 27). The results showed that the number of adverse events among the GLP-1RA group was higher significantly than in the placebo group [RR = 2.3 (1.36, 3.9), *P*-value = 0.002]. The results of the studies were homogenous (*P*-value = 0.23, *I*^2^ = 33%) as presented in Figure 5C.

#### 3.3.11 Serious adverse events

Three studies (22, 23, 27) reported serious adverse events, including severe pneumonia and psychiatric admission, in each group. The overall RR revealed that there was a significant difference among the both groups [RR = 0.44 (0.21, 0.91), *P*-value = 0.03]. The pooled studies were homogenous (*P*-value = 0.94, *I*^2^ = 0%) as presented in Figure 5D.

#### 3.3.12 Bone turnover markers

Two studies (21, 24) assessed the markers of bone turnover through PINP and CTX levels. The influence of exenatide in comparison with placebo was investigated by Eriksson et al. (21). No significant difference was observed between the two groups in terms of both outcomes (*p*-value = 0.27, *p*-value = 0.19; respectively). Also, they followed the participants for up to 3 months and the findings did not reflect a significant difference between the baseline and endpoint values regarding both outcomes (*p*-value = 0.52, *p-*value = 0.93; respectively) (21). Additionally, Maagensen et al. (24) gave liraglutide to the patients and tracked them for 4 months. According to PINP and CTX values, the changes were not significantly different neither between the control and experimental groups (*p*-value = 0.2, *p*-value = 0.07; respectively) nor between start- and end-points in treatment group (*p*-value = 0.49, *p*-value= 0.25; respectively) (24).

## 4. Discussion

The findings of our meta-analysis revealed a significant reduction in body weight, waist circumference, BMI, and serious adverse events among antipsychotic-treated patients with schizophrenia. In addition, the GLP-1RA group was superior to the placebo group in the case of total cholesterol, HbA1c, and blood pressure. However, the overall effect favored placebo over GLP-1RA in terms of GIT adverse events. Though, there was no statistical difference in respiratory side effects, markers of bone turnover, insulin level, and FPG between both groups.

Data from Svensson et al. (26) introduced a significant heterogeneity to the results of body weight, BMI, and waist circumference. Additionally, significant heterogeneity was noticed in the consequences of total cholesterol documented by Larsen et al. (23). This could be elucidated by high variation in the duration of intervention assessment among patients with prediabetes and schizophrenia. The results were not significantly affected after doing the leave-one-out test and excluding the Svensson et al. (26) and Larsen et al. (23) studies from the analysis.

Generally, controlling obesity is a favorable outcome in the management strategy of schizophrenia because it is not only associated with higher risks for insulin resistance, diabetes, and cardiovascular problems but also represents a distressing mental health problem that may reinforce negative self-estimation and treatment compliance (18, 28). The results of this meta-analysis are in the same direction as previous studies' results in terms of body weight, BMI, and waist circumference.

In a previous meta-analysis performed by Zhuo et al. (29) investigated the effect of topiramate and metformin on weight gain in patients treated with antipsychotics. The results of Zhuo et al. (29) showed that topiramate and metformin has significant impact in decreasing body weight and BMI. They reached a conclusion that topiramate had a MD in weight reduction of−3.07 kg by the end of their study, while Metformin's MD was−2.50 kg. As for the BMI, they concluded a result of - 1.59 kg/m2 and 0.61 kg/m2, respectively (29). Compared to our study, we see more potential in our results, as the weight reduction MD is−4.7 and the MD for BMI is−1.65. Interestingly, according to the former studies, GLP-1 RAs were associated with significantly greater weight loss than topiramate or metformin (30–33). Therefore, while topiramate and metformin may be useful adjuncts to lifestyle interventions for weight loss, GLP-1 RAs appear to be more effective at promoting significant and sustained weight loss.

A former meta-analysis conducted by Siskind et al. (18) showed significant lowering effects in FBG in the GLP-1RA group. At the same time, our pooled results along with Wang et al. (19) represented no significant decrease in FBG among the intervention group. This might be explicated by the relatively limited number of included trials Siskind et al. (18). No significant reduction in HbA1c was reported among the GLP-1RA group in the Siskind et al. (18). In contrast, our findings together with Wang et al. (19) showed a significant reduction in HbA1c level in GLP-1RA treated patients. This conflict could be elucidated by variation in baseline characteristics of the population in the included studies in Siskind et al. (18).

Nevertheless, our study is in line with previous reviews in terms of insulin levels between the two groups. The outcomes of glycemic indexes should be taken into consideration because schizophrenic patients are at high risk for growing type 2 diabetes mellitus, they are at 2–5-fold more significant risk for developing diabetes than the general public, though, the exact prevalence varies across studies. Moreover, schizophrenia itself is proposed as the causal factor for DM, and this can be supported by the higher prevalence of diabetes in newly diagnosed young patients even before administering antipsychotic drugs such as clozapine which also rises the chance of increasing type 2 diabetes (34–36).

Although Wang et al. (19) stated superior results in the GLP-1RA group in terms of diastolic blood pressure, the results were not statistically significant in systolic blood pressure. Additionally, the findings of Siskind et al. (18) demonstrated that the total impact did not favor both groups in the case of diastolic and systolic blood pressure. However, our pooled results revealed a significant statistical decrease in systolic and diastolic blood pressure among the GLP-1RA group. This discrepancy might be attributed to the quality of the involved trials and some variations in the population characteristics of the comprised articles.

Regarding total cholesterol, our results showed that GLP-1RA has a significantly favorable role in controlling the total cholesterol level, whereas Wang et al. (19) did not demonstrate a significant difference among both groups. The authors of the Siskind et al. (18) found that the total impact did not favor GLP-1RA over placebo in case of adverse events. Despite there being no statistical difference among both groups in terms of respiratory adverse events, including upper respiratory tract infection and asthma, our findings together with Wang et al. (19) recorded that GLP-1RA was significantly superior to placebo concerning serious adverse events, including severe pneumonia and psychiatric admission. Therefore, the GLP-1RA could be the better choice for alleviating side effects among antipsychotic-treated patients with schizophrenia.

Along with positive and negative symptoms, schizophrenia is characterized by cognitive impairment contributing to the long-term burden associated with the disease and reducing the quality of life (37). Various cognitive domains are impaired in schizophrenia, such as verbal learning, visual learning, memory, problem-solving, attention, and reasoning (38). Therapeutic options for cognitive impairment are still limited as first and second-generation antipsychotics have limited effectiveness in treating cognition (39). Horska et al. (16) showed that the use of GLP-1RAs induces a proposed pro-cognitive effect through improvement of central nervous system deficits which might be also beneficial in schizophrenia patients. GLP-1Rs have binding sites within the cerebral cortex, thalamus, hypothalamus, substantia nigra, circumventricular organ, hippocampus, cerebellum, and brainstem nucleus (40–42). The direct modulatory effects of GLP-1RAs on insulin signaling pathways are associated with improved cognition, as GLP-1RAs activate cyclic adenosine monophosphate (cAMP) (43) which promotes neuroprotection, neuronal development, attenuating oxidative stress and neuroinflammation (44–46). GLP-1RAs might even restore alterations in neurotransmitter function and normalize insulin signaling within the brain, stimulating neuroplasticity (47) also through stimulating the release of brain-derived neurotrophic factor (BDNF), an important factor for learning and memory, low levels of BDNF were previously associated with low cognitive performance in individuals with schizophrenia (48).

Generally, the antipsychotic treatment has been linked to bone fractures risk and osteoporosis (21, 49). Circulating bone turnover markers (BTMs), including PINP and CTX, can be used to evaluate changes in bone formation and resorption (21). According to the former study, GLP-1RA, liraglutide, has been demonstrated to escalate bone formation in body weight-reduced obese women when compared to placebo (50). However, the efficacy of GLP-1RA whether exenatide or liraglutide on bone formation status through markers of bone turnover was still unclear in prediabetes patients with schizophrenia who received clozapine or olanzapine (21, 24). It is essential to highlight those limitations in sample size, the discrepancy in the population characteristics, and the short duration that was conducted to assess the intervention have a critical impact on these findings. Accordingly, we could not pool the results that assessed the influence of GLP-1RA on markers of bone turnover in the meta-analysis model.

### 4.1. Future perspective

GLP-1RA for cardio-metabolic parameters in schizophrenia patients looks promising. Our findings have shown that liraglutide and exenatide can improve glucose metabolism, reduce weight gain, and improve lipid profiles in patients with schizophrenia who are at risk for developing metabolic disorders. Currently, GLP-1RA is administered via subcutaneous injection (51). While this method has been effective in managing diabetes, it can be inconvenient and uncomfortable for patients (51, 52). Therefore, researchers are exploring the possibility of developing an oral form of GLP-1RA (53). This would be more convenient for patients and could potentially improve adherence to treatment. However, there are challenges associated with developing an oral form of GLP-1RA (52–54). The medication would need to survive the acidic environment of the stomach and be absorbed into the bloodstream in a way that maintains its effectiveness (54). Overall, while an oral form of GLP-1RA would be beneficial for patients with type 2 diabetes, more research is needed to develop a safe and effective formulation (53, 54).

On the other hand, it is still unknown if the potential addition of GLP-1RA to second-generation antipsychotic therapies (such as Olanzapine and Clozapine) would eventually positively affect the patient's compliance with the antipsychotic treatment. Metabolic side effects and weight gain are the leading cause of mal-compliance (55). Schizophrenia patients commonly lack insight into various aspects of their illness and the necessary antipsychotic treatment (56), which is an important clinical issue and can be challenging for psychiatrists when patients with psychosis discontinue their antipsychotic treatment due to weight gain, which often leads to new relapse (57) and often new inpatient admission, with resulting healthcare costs (58). In addition, previous studies documented that GLP-1RA, liraglutide, may have potential association with suicidal ideation (59, 60). Therefore, further studies with large sample sizes would be helpful, under careful monitoring of potential suicidal risk.

### 4.2. Limitations and strengths of the study

The major limitations in this study included: (1) a limited number of included trials that assessed the role of GLP-1RA on bone turnover markers in schizophrenia patients, and (2) we observed a marked heterogeneity in some outcomes, which can be accredited to the discrepancy in the period of intervention. Therefore, we recommend further well-designed and high-quality studies with an increased sample size to enhance the possibility of providing level 1 evidence using meta-analysis investigating the efficacy of GLP-1RA on cardiometabolic parameters and bone turnover markers in schizophrenic patients who underwent antipsychotic therapy.

Nevertheless, the strengths of our study are as follows: (1) our meta-analysis represented the last updated evidence assessing the efficacy and safety of GLP-1RA among schizophrenic patients, (2) we provided a more comprehensive analysis in an attempt to solve the previous conflicting findings, (3) we complied the PRISMA checklist when representing this manuscript and conducted all steps as stated in the Cochrane Handbook in our review.

## 5. Conclusions

Ultimately, GLP-1RA appears to be a promising therapeutic candidate, along with their additional neuroprotective effects, through improving insulin signaling, neurotransmission, neuroinflammation, and synaptic plasticity. Nonetheless, the present evidence is not enough to verify the efficacy of GLP-1RA on bone formation status. Accordingly, more trials with an increased sample size are recommended.

## Data availability statement

The original contributions presented in the study are included in the article/Supplementary material, further inquiries can be directed to the corresponding author.

## Author contributions

AK, NM, KA, OA, MH, YY, RT, and SS: conceptualization, data curation, formal analysis, investigation, methodology, visualization, writing—original draft preparation, and writing—review and editing. WH: conceptualization and methodology. ME: supervision, writing—review and editing, proofreading, and visualization. All authors contributed to the article and approved the submitted version.

## Supplementary material

Supplementary Figure S1 Graphical abstract.
