# Supplementary figures and images for: Glucagon-like peptide-1 receptor-agonists treatment for cardio-metabolic parameters in schizophrenia patients: a systematic review and meta-analysis
Source: Front Psychiatry. 2023 May 5;14:1153648. doi: 10.3389/fpsyt.2023.1153648 (PMC10196269; doi:10.3389/fpsyt.2023.1153648)

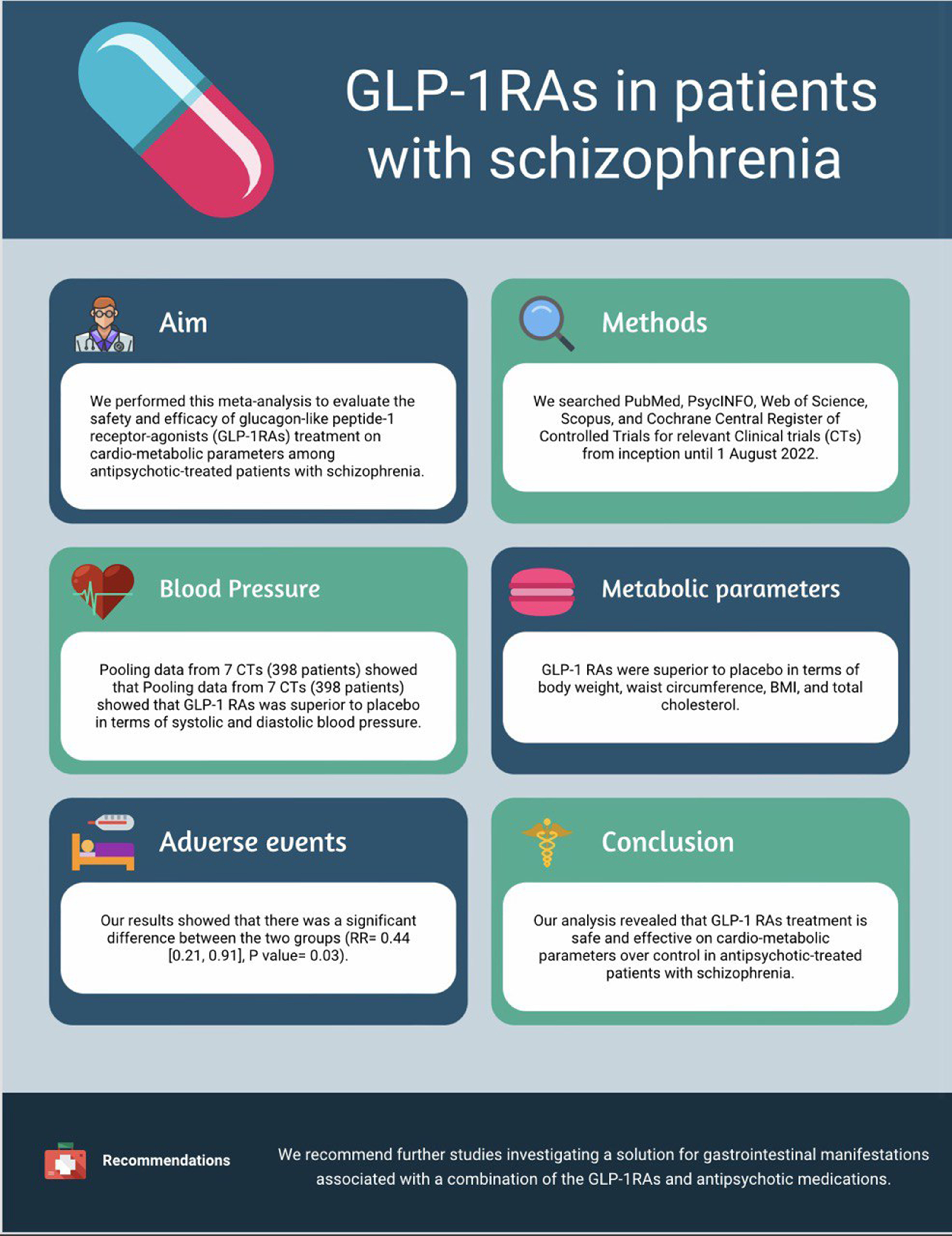

Supplement: Supplementary Figure S1 — Graphical abstract. [file Image_1.JPEG]
